# How Transcription Factor Clusters Shape the Transcriptional Landscape

**DOI:** 10.3390/biom14070875

**Published:** 2024-07-20

**Authors:** Rahul Munshi

**Affiliations:** Joseph Henry Laboratories of Physics and Lewis-Sigler Institute for Integrative Genomics, Princeton University, Princeton, NJ 08544, USA; rahulmun@buffalo.edu

**Keywords:** transcription, gene regulation, biomolecular condensates, transcription factor, clusters, microscopy

## Abstract

In eukaryotic cells, gene transcription typically occurs in discrete periods of promoter activity, interspersed with intervals of inactivity. This pattern deviates from simple stochastic events and warrants a closer examination of the molecular interactions that activate the promoter. Recent studies have identified transcription factor (TF) clusters as key precursors to transcriptional bursting. Often, these TF clusters form at chromatin segments that are physically distant from the promoter, making changes in chromatin conformation crucial for promoter–TF cluster interactions. In this review, I explore the formation and constituents of TF clusters, examining how the dynamic interplay between chromatin architecture and TF clustering influences transcriptional bursting. Additionally, I discuss techniques for visualizing TF clusters and provide an outlook on understanding the remaining gaps in this field.

## 1. Introduction

Regulating gene transcription is central to life. This process involves copying the genetic instructions from a gene’s DNA into a molecule known as messenger ribonucleic acid (mRNA). These mRNA molecules are later translated into proteins [1]. The proper regulation of transcription is crucial for cells to maintain cellular identity during differentiation and homeostasis, and for cell functioning. Eukaryotic transcription involves the recruitment of RNA polymerase II (Pol II) at the promoter region of the DNA. Following transcription initiation [2,3], Pol II facilitates the synthesis of the mRNA molecule by adding RNA nucleotides complementary to the DNA template of the gene. This process is known as transcription elongation [4,5,6,7]. A gene stochastically alternates between states of active transcription (“on” states) and inactive phases (“off” states) where transcription is repressed [8]. The “on” states are frequently marked by “bursts” of activity [9,10,11], during which transcription initiation occurs rapidly, with multiple Pol II molecules simultaneously engaged in transcription elongation [12,13]. Bursting affects the noise in mRNA production within cells, and thus reproducible cell fate and consistent responses to stimuli require additional buffering mechanisms and the spatiotemporal averaging of gene outputs [13,14,15]. Despite the stochastic nature of switching between “on” and “off” states, there are indications of modulation in burst characteristics such as burst duration and burst frequency [13,16,17]. Studies on *Drosophila* developmental genes revealed that a key parameter of regulation comes from the fraction of “on” states [18,19]. This suggests the presence of a shared regulatory mechanism governing the transcription patterns of these genes [19]. Until recently, research predominantly focused on time- and population-averaged relationships between regulatory factors and transcriptional outputs [20]. However, recent discoveries regarding the temporal dynamics of transcription call for efforts to quantify and model dynamic regulatory mechanisms.

The components underlying transcription regulation can be broadly categorized into two factors: cis factors, comprising DNA regulatory elements known as enhancers [21], and trans factors, which consist of protein molecules that interact with these enhancers [22]. The trans factor proteins, such as transcription factor (TF) molecules, bind to enhancer binding sites specifically [23,24,25]. The bound proteins form a complex with other proteins, effectively resulting in the formation of a protein cluster at the enhancer [26,27]. In eukaryotes, DNA base pairs wrap around histone proteins to form nucleosomes, which constitute the fundamental units of chromatin fiber. This chromatin fiber undergoes intricate folding processes, leading to the formation of hierarchical structures [28].

Often, multiple enhancers drive a gene, and these enhancers can be located at various genomic distances, ranging from tens of base pairs to a few megabase pairs of nucleotides away from the promoters [29,30]. Chromatin folding facilitates the formation of loops [31,32], bringing distant enhancers into close proximity, enabling interactions among enhancer-associated protein clusters [33,34]. This complex, comprising enhancers and protein clusters, diffuses [35,36,37] and encounters the gene promoter [38]. Upon encountering it, the protein cluster activates the promoter, inducing a transcriptionally active state [39,40,41,42]. Such a state is marked by the binding of general transcription factors (GTFs) at specific promoter regions, recruiting Pol II to form an assembly of molecules called the transcription pre-initiation complex (PIC) [43,44]. This assembly occurs in steps, leading to the formation of conditions conducive for Pol II to synthesize mRNA molecules [12].

The physical state of interacting enhancer–promoter (E-P) regions along with the protein clusters that govern these interactions [45] is collectively referred to as a “transcription hub” [46,47,48]. Protein molecules such as transcription factors (TFs), cofactors, and mediator molecules [49,50,51] assemble in high concentrations within these hubs, effectively creating a membrane-less organelle [52,53]. When this hub interacts with the promoter, and recruits Pol II, a Pol II cluster may also coincide with this hub [45,54,55,56]. The mechanism of formation of the transcription regulatory cluster, as well as understanding the dynamic regulation of transcription by clusters, has drawn interest for both scientific understanding and therapeutic applications [57,58,59,60,61].

The biochemical assays developed to study the interaction among proteins and nucleotides [62,63,64,65] often fail to capture the dynamics of the clusters resulting from such interactions. Additionally, these assays are typically designed for in vitro analysis and often lack the complexity of physiological conditions of a live cell nucleus [66]. The dynamics of molecules and molecular clusters within the nuclear space unfold at a timescale that microscopy techniques can capture [47,55,67,68]. Hence, biochemical assays are being complemented with fluorescence microscopy-based visualization [56,69,70] as well as light-based probing and perturbation techniques [71,72,73,74,75] in live samples. These techniques entail visualizing fluorescently labeled proteins within live cell nuclei using high-resolution microscopy methods. Through these studies, protein clusters and condensates relevant to transcription were imaged in yeast cells, fruit-fly embryos, and mammalian cell cultures [67,69,76]. Moreover, labeling nascent mRNA transcripts enables the real-time visualization of the transcription process [77,78,79]. Combining these techniques, TF clusters and transcriptional output generation were simultaneously observed in live cells [76,80]. Live quantitative imaging techniques have extended microscopy’s capabilities beyond visualization, revealing the fundamental principles of transcription dynamics [9,18]. Imaging with high spatio-temporal resolution has allowed for the characterization of chromatin dynamics in 3D [81,82,83], enabling the generation of biophysical models that link chromatin folding to transcriptional output [36,82]. Concurrently, researchers have quantified the biophysical properties of TF clusters, such as size, concentration, and total molecular count in live samples [84]. This characterization has shown that the molecular content of the clusters might encode information about the cell’s position within the embryo.

The key to understanding how genes are regulated in real-time is conducting a single-cell study that simultaneously observes cluster dynamics, chromatin dynamics, and transcriptional dynamics for a specific gene. To conduct this real-time analysis, we would need to track various elements, including a chromatin marker indicating the gene promoter, another marker for an enhancer, clusters of assembled transcription factor molecules, and the nascent mRNA at the transcription hotspot. Using high-sensitivity quantitative imaging to observe these labeled species within a gene locus can provide a comprehensive understanding of the temporal relationship among the main components of transcriptional regulation. The beauty of quantitative measurements lies in the insights they yield, which often go beyond empirical causalities and delve into the realm of fundamental relationships rooted in first principles [85,86]. One such fundamental question is how information is transmitted from the transcription factor molecules to the gene promoter [87] via intermediate clustering. While it is evident that clustering dynamically amplifies signals from the nuclear environment at the gene locus, a general model for the comprehension of the spatiotemporal flow of information in gene expression remains elusive.

This review summarizes recent findings on transcriptional regulation through protein clustering in a straightforward language. Despite their various names, all clusters share protein sequestration and compartmentalization characteristics. I will use the term “clustering” to describe this phenomenon and reserve the term “transcriptional hub” for clusters of multiple proteins and chromatin segments that coincide to regulate transcription. The review covers transcription-relevant protein clustering, the interplay of chromatin dynamics and TF clusters in transcriptional bursts, the microscopy techniques enabling these studies, and future research directions in temporal transcriptional regulation.

## 2. Overview of Cluster Formation

TF cluster formation is a complex multi-step process that usually begins when the TF molecules encounter a binding sequence on the DNA. Typically, a DNA binding domain (DBD) of the TF molecule identifies the cognate DNA sequences, spanning 6 to 20 base pairs (bp), within the target enhancers [26,88].

### 2.1. Interaction between TF Molecules and TF Binding Sites in the DNA

**Figure 1:**
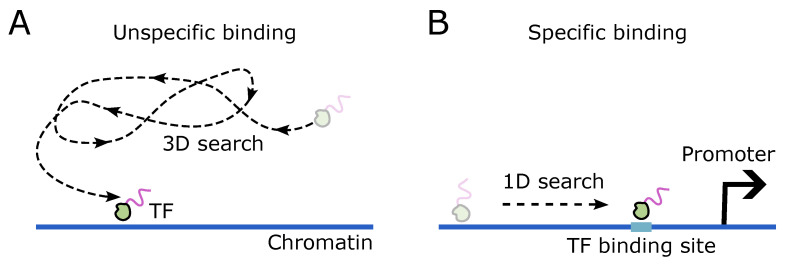
DNA binding site search by the transcription factor (TF). (**A**) Cartoon showing a TF protein undergoing 3D diffusion in the nuclear space to encounter a DNA element. The DNA segment that the TF encounters can be a random site, resulting in an unspecific TF-DNA interaction. (**B**) Representation of a TF protein sliding along the DNA (one-dimensional search) to “find” a cognate binding site.

The TF molecules undergo stochastic diffusion in the 3D nuclear space and occasionally collide with the DNA (Figure 1A). Upon encountering an accessible DNA region, the TF molecule may transiently bind (<1 s) nonspecifically to the DNA. The TF molecule can then scan the DNA for the cognate binding sites by sliding, effectively reducing the search dimension and hence the search time [89,90] (Figure 1B). The weak nature of nonspecific binding facilitates rapid scanning along the DNA [91]. A successful search would result in specific binding, with possibly longer dwell times (>10 s) [89,92]. Overall, the affinities of TF-DNA binding range from low to high [93], with reported binding times spanning from 0.2 to 200 s [94]. It can be argued that only the long-lived bound states have the potential to act as functional bindings leading to cluster formation [95].

**Figure 2:**
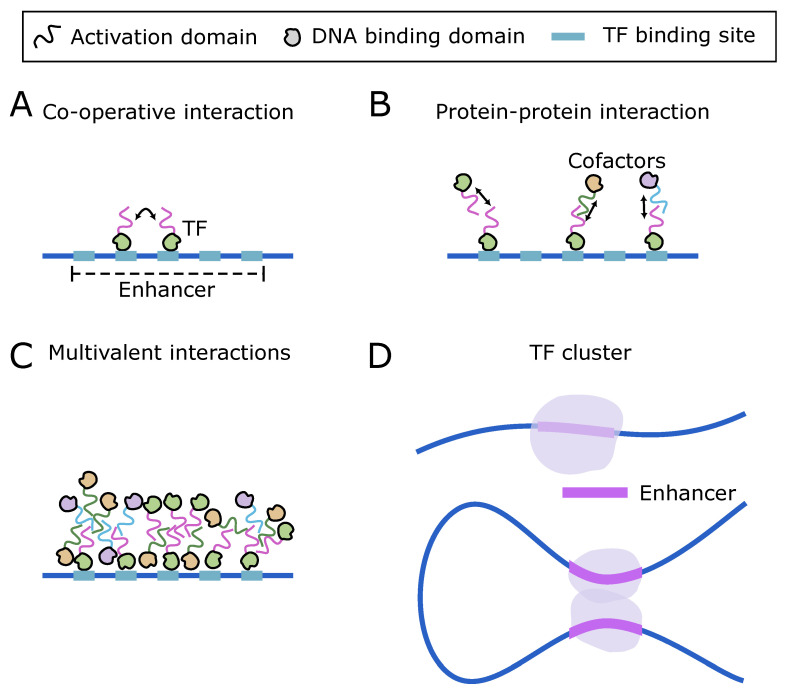
TF cluster formation. (**A**) TF molecules bound to contiguous DNA binding sites in an enhancer may interact cooperatively. (**B**) TF molecules bound to the binding sites in the DNA interact with unbound proteins through their activation domains. (**C**) TF molecules bound to the DNA binding sites in an enhancer interact with each other and with other proteins via their activation domain. (**D**) Interacting proteins in (**C**) form a cluster at the site of the enhancer (**Top**). Protein clusters seeded at two distal DNA segments fuse, creating a bridge between two distant chromatin segments (**Bottom**).

Typically, enhancers consist of several contiguous binding sites [96] that can be cognate to multiple protein species [23]. While such crowding of contiguous binding sites may seem detrimental to binding new molecules, often TFs synergistically assist in recruiting other TF molecules [97] (Figure 2A). Yet another class of transcription factors, known as pioneer factors, aid TF binding by interacting directly with the nucleosomal DNA and facilitate the opening of compacted chromatin [98,99,100]. How pioneer factors access chromatin remains an open question. A theoretical study suggested that pioneer factors accelerate target search more effectively on condensed nucleosomal DNA than on open DNA [47,101]. Additionally, it has been demonstrated that pioneer factors need to bind more transiently than other transcription factors to be effective [102] and that local enrichment might enhance the effectiveness of such transient binding [47].

### 2.2. Interaction of DNA Bound TF with Cofactor Molecules

The simultaneous occupation of neighboring binding sites in the enhancer results in the “trapping” of protein molecules within a confined region (∼100 nm). Distinct from the DBD, TFs typically have an activation domain (AD) [103,104], which frequently contains an intrinsically disordered region (IDR) [104,105]. The IDRs are small motifs that enable the protein to interact with other proteins with high specificity but weak affinity. This characteristic facilitates spontaneous dissociation, allowing for dynamic protein–protein interactions [70,105]. This allows DNA-bound TFs to recruit other proteins, such as coactivators, through interactions mediated by the AD (Figure 2B) [45,106]. The concentration boost reduces the time required for successful searches, resulting in a higher frequency of TF molecules binding to the cognate binding sites in the enhancer [52,107]. Recently, it was proposed that IDR could also influence the TF-DNA binding [108,109].

Thus, regardless of the bound-state dwell time of the TF molecule, the high frequency of binding events would result in a significantly high fraction of enhancer binding site occupation. This stands in stark contrast to scenarios where there is no cooperative interaction among molecules, leading to a lack of local concentration amplification. Such functional synergism has been proposed as a mechanism to explain the potency of low-affinity enhancer targets in driving gene expression [46,67].

The network of self-interactions as well as multivalent interactions among the IDRs within the ADs of the proteins in the neighborhood of enhancers can lead to cluster formation (Figure 2C,D) [110,111] by liquid–liquid phase separation (LLPS) [112,113]. Transcriptionally relevant phase separation has also been observed to be mediated by RNA molecules [114], including long non-coding RNAs [115] Such phase-separated complexes can be distinctly observed at active super-enhancers. Super-enhancers are a group of enhancers that are efficient in recruiting a broad spectrum of cofactors, such as transcriptional activators, chromatin remodelers, and chromatin architectural proteins, and share a common transcription hub among themselves through a network of interacting molecules [116,117]. Indeed, cofactors such as BRD4 and Mediator were found to form phase-separated condensates, stably associated with multiple enhancers simultaneously [118]. Mediator was found to form fairly stable condensates in mouse embryonic stem cells (mESCs), which coincided with Pol II clusters in a transcription-dependent manner [54,69]. This suggests that within a single transcription hub, multiple protein species might be condensed by phase separation.

However, not all proteins that cluster within a transcription hub are necessarily phase-separated [119]. Clusters can also result from an increased local concentration, due to the constrained motion of molecules in the vicinity of enhancers, without undergoing LLPS [97,110]. This phenomenon may arise due to the availability of a high density of binding sites within a short DNA segment in an enhancer [120], often simultaneously activated by chromatin modifiers and pioneer factors [121]. Once bound, the reaction kinetics of TF molecules might also be influenced by cooperative interactions among the TFs, often assisted by nucleosomes [122,123,124]. In such cases, not all molecules interacting with the enhancer may form stable complexes. This is exemplified by the TF Bicoid [125,126] and the pioneer factor Zelda in *Drosophila* embryos. The target enhancers of Bicoid contain Zelda binding sites interspersed with Bicoid binding sites [127,128,129]. However, through cooperative binding [130,131], Bicoid can form stable clusters seeded at the enhancers, while Zelda only transiently enriches the enhancer sites [47], even though the absence of Zelda significantly impacts Bicoid dependent gene expression [47]. Similar findings were observed in the Zelda-mediated activation of Dorsal-dependent gene expression [132]. In another study, Capicua, which acts as a transcriptional repressor in *Drosophila* by interacting with the DNA, was observed to form stable clusters [133], whereas Groucho, which interacts with Capicua in *Drosophila* [134], does not (unpublished). Thus, the constituent molecules in a cluster can have different residence times.

### 2.3. Clusters Confer Information in Nuclear TF Concentration

The significance of a cluster as a non-stoichiometric assembly holds profound implications for precise transcriptional regulation, particularly for developmental patterning genes. These genes depend on enhancers that directly interpret information from the nuclear concentration of transcription factor (TF) molecules. For instance, Bicoid drives transcription in a concentration threshold-dependent manner, with its target gene expressed only in nuclei where Bicoid concentrations exceed a certain threshold [135]. The non-stoichiometric clustering resulting from cooperativity among Bicoid molecules is believed to facilitate this threshold-based action [130,136].

Subsequent research showed that the nuclear concentration of Bicoid is an extremely precise function of the cell’s position in the embryo [85]. Intriguingly, the quantification of Bicoid-dependent gene transcription also revealed remarkable precision in the position dependence of the target gene output [85]. This suggests that enhancers driving target genes can interpret Bicoid concentration with very high accuracy.

A recent study demonstrated that the number of molecules within an average Bicoid cluster accurately represents Bicoid’s nuclear concentrations [84]. A simple explanation for this observation could be that the rate at which Bicoid molecules approach the cluster boundary increases with higher concentration, while the rate at which the molecules escape from the cluster remains constant. This would result in denser clusters, which might facilitate the sustenance of longer transcriptional bursts.

Since Bicoid interacts with several target genes simultaneously, the concentration dependence of an average cluster reflects all such genes, each with varying levels of dependence on Bicoid. How Bicoid clusters related to individual genes interpret concentrations remains to be seen. Nevertheless, TF clustering can serve as an efficient mechanism for the rapid dissemination of nuclear concentration information to the gene locus, which is particularly important for development.

## 3. Interplay of 3D Chromatin Architecture and TF Clusters

The three-dimensional architecture and dynamics of chromatin play a crucial role in the formation of TF clusters. The chromatin is often classified as a polymer, and within short scales, the motion is satisfactorily defined by subdiffusive processes [137,138]. In higher eukaryotes, it is common for multiple genomically distant enhancers to simultaneously regulate a single gene. There have also been observations of the same enhancer regulating multiple genes [29]. In order for enhancers to interact with each other and with the promoter, the chromatin must fold in a way that brings the relevant but distant DNA sections into physical proximity. Folding is the essence of chromatin architecture, and the chromatin folds into hierarchical, unknotted structures, which can be modeled as a fractal globule [139,140]. Genome folding in eukaryotes is not merely a means to pack genetic material of a great linear span into the small 3D space of the cell nucleus. The compaction rather results in the highly organized compartmentalization of the genome, with significant functional implications.

### 3.1. Overview of High-Level 3D Chromatin Architecture

**Figure 3:**
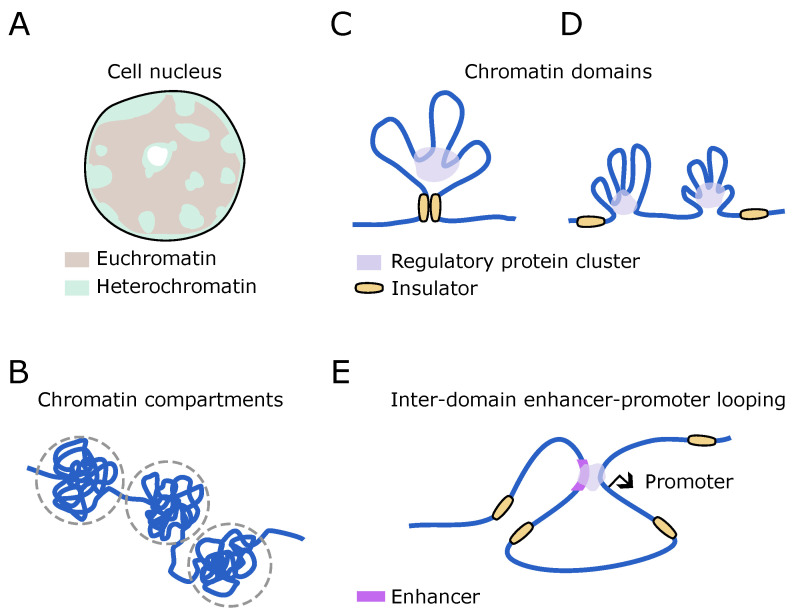
Chromatin organization and topology. (**A**) A schematic of chromatin segregation within the cell nucleus into euchromatin and heterochromatin. The white blob represents a nuclear body. (**B**) Both heterochromatin and euchromatin comprise chromatin compartments (enclosed in grey, dashed circles), which are mutually separated heavily folded sections of the chromosomes. (**C**,**D**) Chromatin conformations within a chromatin domain can include multiple interacting segments regardless of boundary element (insulator) contact. (**E**) Interdomain (inter-TAD) interaction between enhancer and promoter, via a TF cluster.

Classically, the chromosomes are thought to be organized into two distinct territories, heterochromatin and euchromatin. Heterochromatin is the less accessible, transcriptionally inactive region, which is also highly condensed, whereas euchromatin is more accessible, shows histone marks different than heterochromatin, is gene-rich, and is more readily transcribed [141,142,143]. Heterochromatin is predominantly positioned at the nuclear periphery and in the vicinity of nucleoli, whereas euchromatin is located within the nucleus’s interior (Figure 3A) [142]. The compartmentalization results from genome folding and results in the clustering of regions with active genes (A compartments), distinctly separated from regions of inactive genes (B compartments) [144]. Long-range and short-range compartmentalization (Figure 3B) have both been observed to correlate with interacting domains sharing similar transcriptional activity states [145]. Phase separation has been suggested to drive such compartmental segregation [146], among other competing mechanisms [32,147]. The underlying feature of genome organization is the spontaneous folding of the chromatin fiber, and the direct and indirect interaction of chromatin segments with cross-linking proteins [28,148].

### 3.2. Organization of Chromatin Domains

The ensemble average, statistically inferred from cell population data, suggests that the genome is organized into domains characterized by largely the same chromatin and transcriptional state [28]. While the elements within such domains seem to have a high propensity to interact with each other (self-association), interactions with elements outside the domains are generally inhibited (insulation), providing transcriptional regulatory specificity to the domains [149,150,151]. Such domains are often called topologically associated domains (TADs) and can range from a few tens of kilobases to more than a megabase in length [150]. TADs were found to be flanked by distinct boundary elements, which are sometimes referred to as insulators, for their perceived role in insulating the TAD from interaction with neighbors [152,153]. The insulators are characterized as architectural elements, due to the presence of binding sequences for architectural proteins, such as CTCF [154,155]. The precise mechanism by which these boundary elements provide insulation remains unclear, as does the conformation or topology of the chromatin within a TAD. In *Drosophila*, there is evidence of direct pairing between the insulators flanking a TAD, mediated by a bridge of interacting architectural proteins (Figure 3C) [156,157]. A widely accepted model, particularly in vertebrates, proposes that molecular motors load and process along the DNA [158], extruding a loop, that eventually anchors at the boundaries of the TADs [159,160,161]. Microscopy-based single locus studies suggested the existence of multiple extruded loops as the basis of compaction, and hence long-range DNA interactions (Figure 3D) [162], even though live experiments suggest that loops can be both rare and dynamic [82,163].

### 3.3. Long-Range Enhancer–Promoter Association

In addition to intra-TAD loops, loops spanning multiple TADs and connecting specific DNA elements have also been identified [164,165,166]. These loops are typically associated with contacts between enhancers and promoters and are believed to initiate transcriptional activity and thus are functional contacts (Figure 3E) [166,167]. Such focal contacts require the presence of tethering elements that can stabilize long-range stochastic encounters of DNA elements and cannot be solely explained by loop extrusion mechanisms [168]. This can, however, be explained by the Strings and Binders Switch (SBS) model, which treats chromatin as a string with binding sites for molecules. The binding of diffusing molecules to their cognate sites on the string, combined with self-interactions, can lead to the tethering of distant DNA elements and give rise to various stable chromatin architectures [169,170]. Regardless of the model, contacts between DNA elements can serve as loop anchors. However, it is important to note that the apparent contacts observed in population data from sequencing studies do not necessarily indicate real physical contact between DNA elements. Physical contact between DNA segments is untenable as it increases the electrostatic free energy, driven by Coulombic repulsions between the segments [171,172]. To offset this, the charge screening effect, such as that accomplished by the binding of protein molecules, like the TFs, must be attained first [173]. Once the charge has been screened, individual DNA segments can come into close proximity. Indeed, the sustained separation of the *Eve* enhancer and a synthetic promoter (140 kb away), rather than direct contact, was sufficient to trigger transcription in *Drosophila* embryos [83]. Interestingly, this separation distance (∼0.35 μm) was also the closest recorded in the study, suggesting a limitation on how closely an enhancer can approach the promoter. It raises speculation that this closest approach distance may be constrained by the size of an intervening protein cluster. For instance, Bicoid, a key TF in the *Eve* gene regulation, was found to form clusters approximately 400 nm in size [84]. TF binding has also been associated with long-range inter-TAD interactions and with the switching of genome compartments from inactive to active as well [34].

## 4. The Relationship between Clustering and Gene Transcription

**Figure 4:**
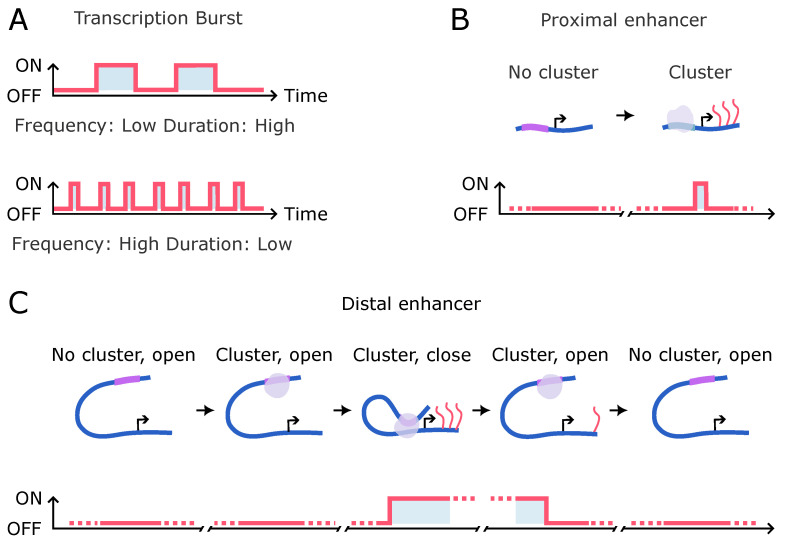
Transcriptional burst regulation by TF clusters. (**A**) Representation of transcriptional burst frequency and duration. The y-axis represents an “on” or “off” state of bursting, while the x-axis denotes time. (**B**) Cartoon showing how the presence or absence of a proximal TF cluster may affect transcriptional bursts. (**C**) Schematic showing how the proximity of a TF cluster bearing enhancer triggers a transcriptional burst. Loss of the TF cluster contact turns the bursting off.

Studies on gene loci where the promoter is genomically distant from the enhancer have consistently shown that the upregulation of transcriptional bursts is anti-correlated with E-P or condensate-promoter distance [54,83,164,174,175]. Although these results indirectly suggest the role of TF clusters in transcriptional bursting, the added complexity of chromatin dynamics complicates the understanding of how TF clusters temporally regulate transcriptional bursting when studying such distal constructs. Conversely, in constructs where the enhancer is genomically proximal to the promoter, an enhancer-seeded TF cluster will consistently be in close physical proximity to the promoter. Hence, temporal fluctuations in cluster intensity can be directly correlated with fluctuations in transcriptional intensity (Figure 4B). Such dynamic relationships between TF cluster fluctuations and transcriptional output were studied in *Drosophila* and yeast [76,80], indicating that TF cluster formation precedes transcriptional bursts.

Understanding transcriptional burst regulation by TF clusters at a molecular level necessitates a deeper comprehension of the phenomenon of bursts (Figure 4A). A transcriptional burst involves the recruitment of multiple Pol II molecules at the gene promoter in rapid succession. This accelerated recruitment facilitates the formation of a Pol II cluster [55,56]. TF clusters can mediate the formation of Pol II clusters through interactions with other molecules [55,118]. Once the promoter accesses the Pol II cluster, the TF cluster seeded at the enhancer no longer needs to actively interact with the promoter during transcriptional elongation. Transcription proceeds as multiple Pol II molecules within the cluster are utilized in succession. When the Pol II molecules in the cluster are depleted, transcription halts, and this “off” state persists until the promoter accesses another Pol II cluster (Figure 4B). This dynamic results in transcriptional bursting, whereby multiple copies of the gene are transcribed rapidly, followed by a period of inactivity [11]. Without a Pol II cluster, stochastic loading of Pol II is expected, leading to the sporadic transcription events rather than sustained bursts.

The impact of the number of molecules in the Pol II cluster on Pol II loading remains unclear. Similarly, the correlation between the number of molecules in the TF cluster and the number of molecules in a Pol II cluster remains uncertain. Therefore, comprehending the temporal regulation of transcriptional bursts by a TF cluster is intricately linked to understanding the precision of information transfer between the TF cluster and the promoter (Figure 4C). We need high-sensitivity quantitative studies of transcribing loci in live cells to delve deeper.

## 5. Studying TF Cluster Dynamics and Transcriptional Bursts

Unraveling the temporal relationships between TF clusters and transcription kinetics and moving beyond mere correlation to establish causation presents a significant challenge. This pursuit requires the incorporation of quantitative live imaging at an extremely high spatio-temporal resolution.

### 5.1. Labeling Proteins and RNA

For imaging, the protein of interest must be labeled with a chromophore that preserves its structural and functional integrity [176,177]. The chromophores must possess suitable photophysical and photochemical properties for the in vivo environment. To achieve an optimal signal-to-noise ratio and minimize photobleaching, the chromophore must exhibit excellent photostability and brightness. Additionally, the excitation/emission spectrum should be carefully selected according to the imaging scheme [178,179]. In recent years, notable advancements have been made in the development of chromophores and molecular tagging technologies [180,181,182,183]. For labeling nascent mRNAs, a commonly employed strategy involves the use of genetically engineered RNA stem-loops, strategically inserted into the non-translating regions of the mRNA [184]. Concurrently, a fluorescent fusion protein, selectively binding to the stem-loops, is genetically introduced. Upon the transcription of the target gene, these stem loops are also transcribed, thereby attracting the labeled fusion proteins. Typically, a series of stem-loops, such as 24 or 48, are incorporated [77,185], allowing for the detection of several fluorescent labels for each nascent mRNA. During a transcriptional burst, multiple nascent mRNAs occupy the transcriptional site, creating a distinct fluorescent hotspot. This transcriptional hotspot serves a dual purpose by marking the relevant transcription sites in the nucleus and facilitating the study of transcriptional dynamics [18,39]. Similarly, proteins of interest can be tagged for live imaging either by genetically fusing a fluorophore to the protein [186] or by genetically expressing protein tags that bind to a ligand introduced into the cells [181,187]. This labeling method allows for the use of fluorescent dyes that are often more photostable and have higher quantum yields compared to traditional fluorescent molecules like green fluorescent protein (GFP) [188].

### 5.2. Imaging Clusters

Microscopy and photo-sensing technologies have developed tremendously over the years [25,189,190,191,192,193,194]. Confocal microscopy, known for its exceptional optical sectioning capabilities compared to traditional widefield microscopes, has provided valuable insights into transcriptional regulation. Recently, the incorporation of a proprietary detector modification, known as the *Airyscan* detector, into Zeiss brand confocal microscopes has enhanced its spatial resolution [195].

Unlike confocal microscopy’s pinhole-based sectioning, two-photon (2P) excitation achieves inherent optical sectioning. This arises from the nonlinear dependence of fluorophore excitation on light intensity, which results in a tightly confined excitation volume at the focal spot of the laser beam. This confined volume minimizes out-of-focus excitation, leading to superior sectioning capability. Utilizing 2P absorption to illuminate samples represents a strategy for confining photon absorption within a small volume. The inherently low absorption cross-section of the 2P signal significantly enhances the signal-to-background ratio, rendering 2P systems advantageous over conventional confocal microscopes for imaging deep tissue signals [196]. When coupled with a high quantum yield photodetector like Gallium Arsenide Phosphide (GaAsP) [197], the signal sensitivity is highly augmented, enabling quantitative studies. This highly localized excitation volume also minimizes photobleaching and phototoxicity within the sample. Despite these advantages, the use of 2P microscopy in the quantitative measurement of transcriptional clusters has been rather limited. Two critical challenges facing two-photon (2P) microscopy are the necessity for highly costly femtosecond infrared lasers and the limited availability of suitable fluorophores compatible with 2P excitation [198].

Frequently, in the pursuit of high-speed volumetric imaging with a high signal-to-background ratio, planar illumination is preferred over point illumination [191,192,193]. The adoption of a scanning “light sheet” instead of a scanning point facilitates accelerated volumetric image acquisition. Moreover, the dispersion of laser power across a plane, rather than its concentration at a point, helps alleviate both phototoxicity and photobleaching. With the advancement of high numerical aperture and high-resolution options [189,199,200], light-sheet microscopes have emerged as invaluable tools in recent inquiries into in vivo protein clusters [69,201].

Various methodologies have been utilized to indirectly investigate protein dynamics by examining the population behavior of proteins and extracting cluster-like features through mathematical fitting and statistical inference. While techniques like Fluorescence Correlation Spectroscopy (FCS) [202,203] and Fluorescence Recovery After Photobleaching (FRAP) [73,204] offer valuable insights into the diffusion characteristics of molecules in cells, they often encounter challenges in accurately capturing the dynamics of molecules within small confined spaces, such as TF clusters. Moreover, the mobility of TF clusters, which diffuse alongside chromatin, poses an additional challenge to their accurate study using these techniques. Single particle tracking has been frequently employed to study the dynamics of molecules within the nuclear environment, providing direct measurements of molecular motion [205]. However, assigning context to the observed characteristics remains a challenge [206]. Super-resolution-based localization microscopy, reliant on the stochastic activation of dye molecules upon exposure to light, furnishes valuable information on cluster lifetime and kinetics [207]. Such techniques have been widely used for cluster studies and have been yielding valuable information.

### 5.3. Application Examples

The optimal microscopy technique should be chosen based on the specific requirements of the study, although in practice, the most readily available option is often utilized. Nevertheless, selecting a superior chromophore can alleviate some of the stringent instrumentation requirements. However, since TF clusters are submicron, often diffraction-limited structures, the spatial resolution requirement for imaging them is extremely high. Another critical consideration is the timescale of the events to be observed. For instance, to correlate TF binding at the gene locus with transcriptional output in yeast, Donovan et al. used HiLo microscopy [76]. To achieve a similar correlation in intact *Drosophila* embryos, Kawasaki et al. employed Airyscan microscopy, which also enabled them to track TF clusters relative to transcriptional hotspots [80]. Additionally, Chen et al. utilized confocal microscopy for the tracking of two chromatin markers relative to the transcriptional hotspot [83]. To study the relative dynamics of protein clusters and chromatin markers together with the transcriptional dynamics, Du et al. employed lattice light sheet microscopy [69]. A two-photon scanning microscope was used by Chen et al. to image GFP-tagged nascent transcripts in live *Drosophila* embryos, enabling the extraction of transcriptional burst parameters at an unprecedented resolution [18]. Mir et al. utilized a lattice light sheet microscope to detect the kinetics and distribution of GFP-tagged TF molecules in *Drosophila* embryos [67,201]. Using stochastically photoactivatable dyes, Cho et al. characterized the lifetimes of clusters and molecular content of Pol II clusters [55].

## 6. Outlook

Technical advancements in understanding DNA–protein interactions [208], protein complex structures [209], chromatin accessibility [210], and chromatin conformation [211] are being increasingly adapted and applied in tandem to unravel the complexities of transcriptional regulation. Among all technical advancements, a significant responsibility for unraveling the complexities of transcriptional regulation by protein clusters falls on optical imaging. The synergistic progression and broad adoption of light microscopy have revealed unprecedented details of dynamic biological processes across scales. The further democratization of imaging systems, combining high spatiotemporal resolution, should be achieved through the commercialization of complex cutting-edge microscopes, which are currently designed, assembled, and operated primarily by specialized optics groups. Such efforts would truly unlock the potential of light microscopes as tools for quantitative measurements rather than merely visualization mediums. Combined with sophisticated protein, RNA, and chromatin tagging techniques, these improvements would greatly enhance our understanding of transcriptional regulation at the molecular level. Each new discovery, while likely to raise further questions visible only at higher resolutions, brings us closer to unraveling the mysteries of the central dogma.

## Abbreviations

The following abbreviations are used in this manuscript:

DNA	Deoxyribonucleic acid
RNA	Ribonucleic acid
TF	Transcription factor
DBD	DNA binding domain
AD	Activation domain
PIC	Pre-initiation complex
GTF	General transcription factor
IDR	Intrinsically disordered region
LLPS	Liquid–liquid phase separation
TAD	Topologically associated domain
SBS	Strings and binders switch
2P	Two-photon
FCS	Fluorescence correlation spectroscopy
FRAP	Fluorescence Recovery After Photobleaching
GFP	Green fluorescent protein

## References

[1] Alberts B., Johnson A., Lewis J., Morgan D., Raff M., Roberts K., Walter P. (2014). Molecular Biology of the Cell.

[2] Haberle V., Stark A. (2018). Eukaryotic core promoters and the functional basis of transcription initiation. Nat. Rev. Mol. Cell Biol..

[3] Larson D.R., Zenklusen D., Wu B., Chao J.A., Singer R.H. (2011). Real-Time Observation of Transcription Initiation and Elongation on an Endogenous Yeast Gene. Science.

[4] Jonkers I., Lis J.T. (2015). Getting up to speed with transcription elongation by RNA polymerase II. Nat. Rev. Mol. Cell Biol..

[5] Chen F.X., Smith E.R., Shilatifard A. (2018). Born to run: Control of transcription elongation by RNA polymerase II. Nat. Rev. Mol. Cell Biol..

[6] Fazal F.M., Meng C.A., Murakami K., Kornberg R.D., Block S.M. (2015). Real-time observation of the initiation of RNA polymerase II transcription. Nature.

[7] Vos S.M. (2021). Understanding transcription across scales: From base pairs to chromosomes. Mol. Cell.

[8] Raj A., Oudenaarden A.V. (2008). Nature, Nurture, or Chance: Stochastic Gene Expression and Its Consequences. Cell.

[9] Raj A., Peskin C.S., Tranchina D., Vargas D.Y., Tyagi S. (2006). Stochastic mRNA Synthesis in Mammalian Cells. PLoS Biol..

[10] Tunnacliffe E., Chubb J.R. (2020). What Is a Transcriptional Burst?. Trends Genet..

[11] Meeussen J.V., Lenstra T.L. (2024). Time will tell: Comparing timescales to gain insight into transcriptional bursting. Trends Genet..

[12] Sainsbury S., Bernecky C., Cramer P. (2015). Structural basis of transcription initiation by RNA polymerase II. Nat. Rev. Mol. Cell Biol..

[13] Porello E.A.L., Trudeau R.T., Lim B. (2023). Transcriptional bursting: Stochasticity in deterministic development. Development.

[14] Urban E.A., Johnston R.J. (2018). Buffering and Amplifying Transcriptional Noise During Cell Fate Specification. Front. Genet..

[15] Little S., Tikhonov M., Gregor T. (2013). Precise Developmental Gene Expression Arises from Globally Stochastic Transcriptional Activity. Cell.

[16] Kumar N., Singh A., Kulkarni R.V. (2015). Transcriptional Bursting in Gene Expression: Analytical Results for General Stochastic Models. PLoS Comput. Biol..

[17] Dar R.D., Razooky B.S., Singh A., Trimeloni T.V., McCollum J.M., Cox C.D., Simpson M.L., Weinberger L.S. (2012). Transcriptional burst frequency and burst size are equally modulated across the human genome. Proc. Natl. Acad. Sci. USA.

[18] Chen P.T., Zoller B., Levo M., Gregor T. (2023). Common bursting relationships underlie eukaryotic transcription dynamics. arXiv.

[19] Zoller B., Little S.C., Gregor T. (2018). Diverse Spatial Expression Patterns Emerge from Unified Kinetics of Transcriptional Bursting. Cell.

[20] Xu H., Sepúlveda L.A., Figard L., Sokac A.M., Golding I. (2015). Combining protein and mRNA quantification to decipher transcriptional regulation. Nat. Methods.

[21] Benabdallah N.S., Bickmore W.A. (2015). Regulatory Domains and Their Mechanisms. Cold Spring Harb. Symp. Quant. Biol..

[22] Giammartino D.C.D., Polyzos A., Apostolou E. (2020). Transcription factors: Building hubs in the 3D space. Cell Cycle.

[23] Spitz F., Furlong E.E.M. (2012). Transcription factors: From enhancer binding to developmental control. Nat. Rev. Genet..

[24] Rohs R., Jin X., West S.M., Joshi R., Honig B., Mann R.S. (2010). Origins of Specificity in Protein-DNA Recognition. Annu. Rev. Biochem..

[25] Gebhardt J.C.M., Suter D.M., Roy R., Zhao Z.W., Chapman A.R., Basu S., Maniatis T., Xie X.S. (2013). Single-molecule imaging of transcription factor binding to DNA in live mammalian cells. Nat. Methods.

[26] Ptashne M., Gann A. (1997). Transcriptional activation by recruitment. Nature.

[27] Bhat P., Honson D., Guttman M. (2021). Nuclear compartmentalization as a mechanism of quantitative control of gene expression. Nat. Rev. Mol. Cell Biol..

[28] Misteli T. (2020). The Self-Organizing Genome: Principles of Genome Architecture and Function. Cell.

[29] Furlong E.E.M., Levine M. (2018). Developmental enhancers and chromosome topology. Science.

[30] Laat W.d., Duboule D. (2013). Topology of mammalian developmental enhancers and their regulatory landscapes. Nature.

[31] Deng W., Lee J., Wang H., Miller J., Reik A., Gregory P., Dean A., Blobel G. (2012). Controlling Long-Range Genomic Interactions at a Native Locus by Targeted Tethering of a Looping Factor. Cell.

[32] Miele A., Dekker J. (2008). Long-range chromosomal interactions and gene regulation. Mol. Biosyst..

[33] Kim S., Shendure J. (2019). Mechanisms of Interplay between Transcription Factors and the 3D Genome. Mol. Cell.

[34] Stadhouders R., Filion G.J., Graf T. (2019). Transcription factors and 3D genome conformation in cell-fate decisions. Nature.

[35] Popay T.M., Dixon J.R. (2022). Coming full circle: On the origin and evolution of the looping model for enhancer—Promoter communication. J. Biol. Chem..

[36] Brückner D.B., Chen H., Barinov L., Zoller B., Gregor T. (2023). Stochastic motion and transcriptional dynamics of pairs of distal DNA loci on a compacted chromosome. Science.

[37] Lampo T., Kennard A., Spakowitz A. (2016). Physical Modeling of Dynamic Coupling between Chromosomal Loci. Biophys. J..

[38] Yamamoto T., Sakaue T., Schiessel H. (2021). Slow chromatin dynamics enhances promoter accessibility to transcriptional condensates. Nucleic Acids Res..

[39] Fukaya T., Lim B., Levine M. (2016). Enhancer Control of Transcriptional Bursting. Cell.

[40] Schoenfelder S., Fraser P. (2019). Long-range enhancer—Promoter contacts in gene expression control. Nat. Rev. Genet..

[41] Vernimmen D., Gobbi M.D., Sloane-Stanley J.A., Wood W.G., Higgs D.R. (2007). Long-range chromosomal interactions regulate the timing of the transition between poised and active gene expression. EMBO J..

[42] Stadhouders R., Vidal E., Serra F., Stefano B.D., Dily F.L., Quilez J., Gomez A., Collombet S., Berenguer C., Cuartero Y. (2018). Transcription factors orchestrate dynamic interplay between genome topology and gene regulation during cell reprogramming. Nat. Genet..

[43] Orphanides G., Lagrange T., Reinberg D. (1996). The general transcription factors of RNA polymerase II. Genes Dev..

[44] Malik S., Roeder R.G. (2023). Regulation of the RNA polymerase II pre-initiation complex by its associated coactivators. Nat. Rev. Genet..

[45] Boija A., Klein I.A., Sabari B.R., Dall’Agnese A., Coffey E.L., Zamudio A.V., Li C.H., Shrinivas K., Manteiga J.C., Hannett N.M. (2018). Transcription Factors Activate Genes through the Phase-Separation Capacity of Their Activation Domains. Cell.

[46] Tsai A., Muthusamy A.K., Alves M.R., Lavis L.D., Singer R.H., Stern D.L., Crocker J. (2017). Nuclear microenvironments modulate transcription from low-affinity enhancers. eLife.

[47] Hayward-Lara G., Fischer M.D., Mir M. (2024). Dynamic microenvironments shape nuclear organization and gene expression. Curr. Opin. Genet. Dev..

[48] Zhu I., Song W., Ovcharenko I., Landsman D. (2021). A model of active transcription hubs that unifies the roles of active promoters and enhancers. Nucleic Acids Res..

[49] Allen B.L., Taatjes D.J. (2015). The Mediator complex: A central integrator of transcription. Nat. Rev. Mol. Cell Biol..

[50] Khattabi L.E., Zhao H., Kalchschmidt J., Young N., Jung S., Blerkom P.V., Kieffer-Kwon P., Kieffer-Kwon K.R., Park S., Wang X. (2019). A Pliable Mediator Acts as a Functional Rather Than an Architectural Bridge between Promoters and Enhancers. Cell.

[51] Palacio M., Taatjes D.J. (2022). Merging Established Mechanisms with New Insights: Condensates, Hubs, and the Regulation of RNA Polymerase II Transcription. J. Mol. Biol..

[52] Banani S.F., Lee H.O., Hyman A.A., Rosen M.K. (2017). Biomolecular condensates: Organizers of cellular biochemistry. Nat. Rev. Mol. Cell Biol..

[53] Gomes E., Shorter J. (2019). The molecular language of membraneless organelles. J. Biol. Chem..

[54] Cho W.K., Spille J.H., Hecht M., Lee C., Li C., Grube V., Cisse I.I. (2018). Mediator and RNA polymerase II clusters associate in transcription-dependent condensates. Science.

[55] Cho W.K., Jayanth N., English B.P., Inoue T., Andrews J.O., Conway W., Grimm J.B., Spille J.H., Lavis L.D., Lionnet T. (2016). RNA Polymerase II cluster dynamics predict mRNA output in living cells. eLife.

[56] Cisse I.I., Izeddin I., Causse S.Z., Boudarene L., Senecal A., Muresan L., Dugast-Darzacq C., Hajj B., Dahan M., Darzacq X. (2013). Real-Time Dynamics of RNA Polymerase II Clustering in Live Human Cells. Science.

[57] McSwiggen D.T., Hansen A.S., Teves S.S., Marie-Nelly H., Hao Y., Heckert A.B., Umemoto K.K., Dugast-Darzacq C., Tjian R., Darzacq X. (2019). Evidence for DNA-mediated nuclear compartmentalization distinct from phase separation. eLife.

[58] Mittag T., Pappu R.V. (2022). A conceptual framework for understanding phase separation and addressing open questions and challenges. Mol. Cell.

[59] Chen H., Pugh B.F. (2021). What do Transcription Factors Interact With?. J. Mol. Biol..

[60] Suter D.M. (2020). Transcription Factors and DNA Play Hide and Seek. Trends Cell Biol..

[61] Ryu K., Park G., Cho W.K. (2024). Emerging insights into transcriptional condensates. Exp. Mol. Med..

[62] Park P.J. (2009). ChIP—Seq: Advantages and challenges of a maturing technology. Nat. Rev. Genet..

[63] Buenrostro J.D., Wu B., Chang H.Y., Greenleaf W.J. (2015). ATAC-seq: A Method for Assaying Chromatin Accessibility Genome-Wide. Curr. Protoc. Mol. Biol..

[64] Alberti S., Saha S., Woodruff J.B., Franzmann T.M., Wang J., Hyman A.A. (2018). A User’s Guide for Phase Separation Assays with Purified Proteins. J. Mol. Biol..

[65] Ren B., Robert F., Wyrick J.J., Aparicio O., Jennings E.G., Simon I., Zeitlinger J., Schreiber J., Hannett N., Kanin E. (2000). Genome-Wide Location and Function of DNA Binding Proteins. Science.

[66] Irgen-Gioro S., Yoshida S., Walling V., Chong S. (2022). Fixation can change the appearance of phase separation in living cells. eLife.

[67] Mir M., Reimer A., Haines J.E., Li X.Y., Stadler M., Garcia H., Eisen M.B., Darzacq X. (2017). Dense Bicoid hubs accentuate binding along the morphogen gradient. Genes Dev..

[68] Izeddin I., Récamier V., Bosanac L., Cissé I.I., Boudarene L., Dugast-Darzacq C., Proux F., Bénichou O., Voituriez R., Bensaude O. (2014). Single-molecule tracking in live cells reveals distinct target-search strategies of transcription factors in the nucleus. eLife.

[69] Du M., Stitzinger S.H., Spille J.H., Cho W.K., Lee C., Hijaz M., Quintana A., Cissé I.I. (2024). Direct observation of a condensate effect on super-enhancer controlled gene bursting. Cell.

[70] Chong S., Dugast-Darzacq C., Liu Z., Dong P., Dailey G.M., Cattoglio C., Heckert A., Banala S., Lavis L., Darzacq X. (2018). Imaging dynamic and selective low-complexity domain interactions that control gene transcription. Science.

[71] Kim Y.J., Lee M., Lee Y.T., Jing J., Sanders J.T., Botten G.A., He L., Lyu J., Zhang Y., Mettlen M. (2023). Light-activated macromolecular phase separation modulates transcription by reconfiguring chromatin interactions. Sci. Adv..

[72] Lee M., Moon H.C., Jeong H., Kim D.W., Park H.Y., Shin Y. (2024). Optogenetic control of mRNA condensation reveals an intimate link between condensate material properties and functions. Nat. Commun..

[73] Taylor N.O., Wei M.T., Stone H.A., Brangwynne C.P. (2019). Quantifying Dynamics in Phase-Separated Condensates Using Fluorescence Recovery after Photobleaching. Biophys. J..

[74] Shimobayashi S.F., Ronceray P., Sanders D.W., Haataja M.P., Brangwynne C.P. (2021). Nucleation landscape of biomolecular condensates. Nature.

[75] Shin Y., Berry J., Pannucci N., Haataja M.P., Toettcher J.E., Brangwynne C.P. (2017). Spatiotemporal Control of Intracellular Phase Transitions Using Light-Activated optoDroplets. Cell.

[76] Donovan B.T., Huynh A., Ball D.A., Patel H.P., Poirier M.G., Larson D.R., Ferguson M.L., Lenstra T.L. (2019). Live-cell imaging reveals the interplay between transcription factors, nucleosomes, and bursting. EMBO J..

[77] Garcia H., Tikhonov M., Lin A., Gregor T. (2013). Quantitative Imaging of Transcription in Living *Drosophila* Embryos Links Polymerase Activity to Patterning. Curr. Biol..

[78] Golding I., Paulsson J., Zawilski S.M., Cox E.C. (2005). Real-Time Kinetics of Gene Activity in Individual Bacteria. Cell.

[79] Yunger S., Rosenfeld L., Garini Y., Shav-Tal Y. (2010). Single-allele analysis of transcription kinetics in living mammalian cells. Nat. Methods.

[80] Kawasaki K., Fukaya T. (2023). Functional coordination between transcription factor clustering and gene activity. Mol. Cell.

[81] Bintu B., Mateo L.J., Su J.H., Sinnott-Armstrong N.A., Parker M., Kinrot S., Yamaya K., Boettiger A.N., Zhuang X. (2018). Super-resolution chromatin tracing reveals domains and cooperative interactions in single cells. Science.

[82] Gabriele M., Brandão H.B., Grosse-Holz S., Jha A., Dailey G.M., Cattoglio C., Hsieh T.H.S., Mirny L., Zechner C., Hansen A.S. (2022). Dynamics of CTCF- and cohesin-mediated chromatin looping revealed by live-cell imaging. Science.

[83] Chen H., Levo M., Barinov L., Fujioka M., Jaynes J.B., Gregor T. (2018). Dynamic interplay between enhancer—Promoter topology and gene activity. Nat. Genet..

[84] Munshi R., Ling J., Ryabichko S., Wieschaus E.F., Gregor T. (2024). Transcription factor clusters as information transfer agents. arXiv.

[85] Gregor T., Tank D.W., Wieschaus E.F., Bialek W. (2007). Probing the Limits to Positional Information. Cell.

[86] Petkova M.D., Tkačik G., Bialek W., Wieschaus E.F., Gregor T. (2019). Optimal Decoding of Cellular Identities in a Genetic Network. Cell.

[87] Tkačik G., Callan C.G., Bialek W. (2008). Information flow and optimization in transcriptional regulation. Proc. Natl. Acad. Sci. USA.

[88] Bintu L., Buchler N.E., Garcia H.G., Gerland U., Hwa T., Kondev J., Phillips R. (2005). Transcriptional regulation by the numbers: Models. Curr. Opin. Genet. Dev..

[89] Hippel P.H.v., Berg O.G. (1989). Facilitated Target Location in Biological Systems. J. Biol. Chem..

[90] Mirny L., Slutsky M., Wunderlich Z., Tafvizi A., Leith J., Kosmrlj A. (2009). How a protein searches for its site on DNA: The mechanism of facilitated diffusion. J. Phys. A Math. Theor..

[91] Marklund E., Oosten B.v., Mao G., Amselem E., Kipper K., Sabantsev A., Emmerich A., Globisch D., Zheng X., Lehmann L.C. (2020). DNA surface exploration and operator bypassing during target search. Nature.

[92] Chen J., Zhang Z., Li L., Chen B.C., Revyakin A., Hajj B., Legant W., Dahan M., Lionnet T., Betzig E. (2014). Single-Molecule Dynamics of Enhanceosome Assembly in Embryonic Stem Cells. Cell.

[93] Rastogi C., Rube H.T., Kribelbauer J.F., Crocker J., Loker R.E., Martini G.D., Laptenko O., Freed-Pastor W.A., Prives C., Stern D.L. (2018). Accurate and sensitive quantification of protein-DNA binding affinity. Proc. Natl. Acad. Sci. USA.

[94] Geertz M., Shore D., Maerkl S.J. (2012). Massively parallel measurements of molecular interaction kinetics on a microfluidic platform. Proc. Natl. Acad. Sci. USA.

[95] Garcia D.A., Fettweis G., Presman D.M., Paakinaho V., Jarzynski C., Upadhyaya A., Hager G. (2021). Power-law behavior of transcription factor dynamics at the single-molecule level implies a continuum affinity model. Nucleic Acids Res..

[96] Berman B.P., Nibu Y., Pfeiffer B.D., Tomancak P., Celniker S.E., Levine M., Rubin G.M., Eisen M.B. (2002). Exploiting transcription factor binding site clustering to identify cis-regulatory modules involved in pattern formation in the *Drosophila* genome. Proc. Natl. Acad. Sci. USA.

[97] Wagh K., Stavreva D.A., Upadhyaya A., Hager G.L. (2023). Transcription Factor Dynamics: One Molecule at a Time. Annu. Rev. Cell Dev. Biol..

[98] Zaret K.S. (2020). Pioneer Transcription Factors Initiating Gene Network Changes. Annu. Rev. Genet..

[99] Zaret K.S., Carroll J.S. (2011). Pioneer transcription factors: Establishing competence for gene expression. Genes Dev..

[100] Zaret K.S., Mango S.E. (2016). Pioneer transcription factors, chromatin dynamics, and cell fate control. Curr. Opin. Genet. Dev..

[101] Felipe C., Shin J., Kolomeisky A.B. (2022). How Pioneer Transcription Factors Search for Target Sites on Nucleosomal DNA. J. Phys. Chem. B.

[102] Donovan B.T., Chen H., Jipa C., Bai L., Poirier M.G. (2019). Dissociation rate compensation mechanism for budding yeast pioneer transcription factors. eLife.

[103] Udupa A., Kotha S.R., Staller M.V. (2024). Commonly asked questions about transcriptional activation domains. Curr. Opin. Struct. Biol..

[104] Soto L.F., Li Z., Santoso C.S., Berenson A., Ho I., Shen V.X., Yuan S., Bass J.I.F. (2022). Compendium of human transcription factor effector domains. Mol. Cell.

[105] Dyson H.J., Wright P.E. (2016). Role of Intrinsic Protein Disorder in the Function and Interactions of the Transcriptional Coactivators CREB-binding Protein (CBP) and p300*. J. Biol. Chem..

[106] Garcia D.A., Johnson T.A., Presman D.M., Fettweis G., Wagh K., Rinaldi L., Stavreva D.A., Paakinaho V., Jensen R.A., Mandrup S. (2021). An intrinsically disordered region-mediated confinement state contributes to the dynamics and function of transcription factors. Mol. Cell.

[107] Kent S., Brown K., Yang C.h., Alsaihati N., Tian C., Wang H., Ren X. (2020). Phase-Separated Transcriptional Condensates Accelerate Target-Search Process Revealed by Live-Cell Single-Molecule Imaging. Cell Rep..

[108] Chappleboim M., Naveh-Tassa S., Carmi M., Levy Y., Barkai N. (2024). Ordered and disordered regions of the Origin Recognition Complex direct differential in vivo binding at distinct motif sequences. Nucleic Acids Res..

[109] Hurieva B., Kumar D.K., Morag R., Lupo O., Carmi M., Barkai N., Jonas F. (2024). Disordered sequences of transcription factors regulate genomic binding by integrating diverse sequence grammars and interaction types. Nucleic Acids Res..

[110] Lu F., Lionnet T. (2021). Transcription Factor Dynamics. Cold Spring Harb. Perspect. Biol..

[111] Meeussen J.V.W., Pomp W., Brouwer I., de Jonge W.J., Patel H.P., Lenstra T.L. (2023). Transcription factor clusters enable target search but do not contribute to target gene activation. Nucleic Acids Res..

[112] Shrinivas K., Sabari B.R., Coffey E.L., Klein I.A., Boija A., Zamudio A.V., Schuijers J., Hannett N.M., Sharp P.A., Young R.A. (2019). Enhancer Features that Drive Formation of Transcriptional Condensates. Mol. Cell.

[113] Hnisz D., Shrinivas K., Young R.A., Chakraborty A.K., Sharp P.A. (2017). A Phase Separation Model for Transcriptional Control. Cell.

[114] Henninger J.E., Oksuz O., Shrinivas K., Sagi I., LeRoy G., Zheng M.M., Andrews J.O., Zamudio A.V., Lazaris C., Hannett N.M. (2021). RNA-Mediated Feedback Control of Transcriptional Condensates. Cell.

[115] Mattick J.S., Amaral P.P., Carninci P., Carpenter S., Chang H.Y., Chen L.L., Chen R., Dean C., Dinger M.E., Fitzgerald K.A. (2023). Long non-coding RNAs: Definitions, functions, challenges and recommendations. Nat. Rev. Mol. Cell Biol..

[116] Pott S., Lieb J.D. (2015). What are super-enhancers?. Nat. Genet..

[117] Hnisz D., Abraham B., Lee T., Lau A., Saint-André V., Sigova A., Hoke H., Young R. (2013). Super-Enhancers in the Control of Cell Identity and Disease. Cell.

[118] Sabari B.R., Dall’Agnese A., Boija A., Klein I.A., Coffey E.L., Shrinivas K., Abraham B.J., Hannett N.M., Zamudio A.V., Manteiga J.C. (2018). Coactivator condensation at super-enhancers links phase separation and gene control. Science.

[119] Trojanowski J., Frank L., Rademacher A., Mücke N., Grigaitis P., Rippe K. (2022). Transcription activation is enhanced by multivalent interactions independent of phase separation. Mol. Cell.

[120] Arnone M.I., Davidson E.H. (1997). The hardwiring of development: Organization and function of genomic regulatory systems. Development.

[121] Calo E., Wysocka J. (2013). Modification of Enhancer Chromatin: What, How, and Why?. Mol. Cell.

[122] Rao S., Ahmad K., Ramachandran S. (2021). Cooperative binding between distant transcription factors is a hallmark of active enhancers. Mol. Cell.

[123] Mirny L.A. (2010). Nucleosome-mediated cooperativity between transcription factors. Proc. Natl. Acad. Sci. USA.

[124] Morgunova E., Taipale J. (2017). Structural perspective of cooperative transcription factor binding. Curr. Opin. Struct. Biol..

[125] Driever W., Nüsslein-Volhard C. (1988). A gradient of bicoid protein in *Drosophila* embryos. Cell.

[126] Driever W., Nüsslein-Volhard C. (1988). The bicoid protein determines position in the *Drosophila* embryo in a concentration-dependent manner. Cell.

[127] Blythe S.A., Wieschaus E.F. (2016). Establishment and maintenance of heritable chromatin structure during early *Drosophila* embryogenesis. eLife.

[128] Ling J., Umezawa K.Y., Scott T., Small S. (2019). Bicoid-Dependent Activation of the Target Gene hunchback Requires a Two-Motif Sequence Code in a Specific Basal Promoter. Mol. Cell.

[129] Xu Z., Chen H., Ling J., Yu D., Struffi P., Small S. (2014). Impacts of the ubiquitous factor Zelda on Bicoid-dependent DNA binding and transcription in *Drosophila*. Genes Dev..

[130] Burz D.S., Rivera-Pomar R., Jäckle H., Hanes S.D. (1998). Cooperative DNA-binding by Bicoid provides a mechanism for threshold-dependent gene activation in the *Drosophila* embryo. EMBO J..

[131] Lebrecht D., Foehr M., Smith E., Lopes F.J.P., Vanario-Alonso C.E., Reinitz J., Burz D.S., Hanes S.D. (2005). Bicoid cooperative DNA binding is critical for embryonic patterning in *Drosophila*. Proc. Natl. Acad. Sci. USA.

[132] Yamada S., Whitney P.H., Huang S.K., Eck E.C., Garcia H.G., Rushlow C.A. (2019). The *Drosophila* Pioneer Factor Zelda Modulates the Nuclear Microenvironment of a Dorsal Target Enhancer to Potentiate Transcriptional Output. Curr. Biol..

[133] Zhang L., Hodgins L., Sakib S., Mahmood A., Perez-Romero C., Marmion R.A., Dostatni N., Fradin C. (2024). Both the transcriptional activator, Bcd, and transcriptional repressor, Cic, form small mobile oligomeric clusters in early fly embryo nuclei. bioRxiv.

[134] Forés M., Ajuria L., Samper N., Astigarraga S., Nieva C., Grossman R., González-Crespo S., Paroush Z., Jiménez G. (2015). Origins of Context-Dependent Gene Repression by Capicua. PLoS Genet..

[135] Driever W., Nüsslein-Volhard C. (1989). The bicoid protein is a positive regulator of hunchback transcription in the early *Drosophila* embryo. Nature.

[136] Singh A.P., Wu P., Ryabichko S., Raimundo J., Swan M., Wieschaus E., Gregor T., Toettcher J.E. (2022). Optogenetic control of the Bicoid morphogen reveals fast and slow modes of gap gene regulation. Cell Rep..

[137] Weber S.C., Theriot J.A., Spakowitz A.J. (2010). Subdiffusive motion of a polymer composed of subdiffusive monomers. Phys. Rev. E.

[138] Câmara A.S., Mascher M. (2023). Consistencies and contradictions in different polymer models of chromatin architecture. Comput. Struct. Biotechnol. J..

[139] Grosberg A., Rabin Y., Havlin S., Neer A. (1993). Crumpled Globule Model of the Three-Dimensional Structure of DNA. EPL (Europhys. Lett.).

[140] Mirny L.A. (2011). The fractal globule as a model of chromatin architecture in the cell. Chromosome Res..

[141] Huisinga K.L., Brower-Toland B., Elgin S.C.R. (2006). The contradictory definitions of heterochromatin: Transcription and silencing. Chromosoma.

[142] Bickmore W., van Steensel B. (2013). Genome Architecture: Domain Organization of Interphase Chromosomes. Cell.

[143] Talbert P.B., Henikoff S. (2017). Histone variants on the move: Substrates for chromatin dynamics. Nat. Rev. Mol. Cell Biol..

[144] Lieberman-Aiden E., Berkum N.L.v., Williams L., Imakaev M., Ragoczy T., Telling A., Amit I., Lajoie B.R., Sabo P.J., Dorschner M.O. (2009). Comprehensive Mapping of Long-Range Interactions Reveals Folding Principles of the Human Genome. Science.

[145] Rowley M.J., Nichols M.H., Lyu X., Ando-Kuri M., Rivera I.S.M., Hermetz K., Wang P., Ruan Y., Corces V.G. (2017). Evolutionarily Conserved Principles Predict 3D Chromatin Organization. Mol. Cell.

[146] Erdel F., Rippe K. (2018). Formation of Chromatin Subcompartments by Phase Separation. Biophys. J..

[147] McSwiggen D.T., Mir M., Darzacq X., Tjian R. (2019). Evaluating phase separation in live cells: Diagnosis, caveats, and functional consequences. Genes Dev..

[148] Hildebrand E.M., Dekker J. (2020). Mechanisms and Functions of Chromosome Compartmentalization. Trends Biochem. Sci..

[149] Rowley M.J., Corces V.G. (2018). Organizational principles of 3D genome architecture. Nat. Rev. Genet..

[150] Szabo Q., Bantignies F., Cavalli G. (2019). Principles of genome folding into topologically associating domains. Sci. Adv..

[151] Dixon J.R., Selvaraj S., Yue F., Kim A., Li Y., Shen Y., Hu M., Liu J.S., Ren B. (2012). Topological domains in mammalian genomes identified by analysis of chromatin interactions. Nature.

[152] Dixon J., Gorkin D., Ren B. (2016). Chromatin Domains: The Unit of Chromosome Organization. Mol. Cell.

[153] Brasset E., Vaury C. (2005). Insulators are fundamental components of the eukaryotic genomes. Heredity.

[154] Valenzuela L., Kamakaka R.T. (2006). Chromatin Insulators*. Genetics.

[155] Kuhn E.J., Geyer P.K. (2003). Genomic insulators: Connecting properties to mechanism. Curr. Opin. Cell Biol..

[156] Fujioka M., Mistry H., Schedl P., Jaynes J.B. (2016). Determinants of Chromosome Architecture: Insulator Pairing in cis and in trans. PLoS Genet..

[157] Eagen K.P., Aiden E.L., Kornberg R.D. (2017). Polycomb-mediated chromatin loops revealed by a subkilobase-resolution chromatin interaction map. Proc. Natl. Acad. Sci. USA.

[158] Davidson I.F., Goetz D., Zaczek M.P., Molodtsov M.I., Veld P.J.H.i.t., Weissmann F., Litos G., Cisneros D.A., Ocampo-Hafalla M., Ladurner R. (2016). Rapid movement and transcriptional re-localization of human cohesin on DNA. EMBO J..

[159] Beagan J.A., Phillips-Cremins J.E. (2020). On the existence and functionality of topologically associating domains. Nat. Genet..

[160] Phillips J.E., Corces V.G. (2009). CTCF: Master Weaver of the Genome. Cell.

[161] Goloborodko A., Marko J., Mirny L. (2016). Chromosome Compaction by Active Loop Extrusion. Biophys. J..

[162] Chen L.F., Long H.K., Park M., Swigut T., Boettiger A.N., Wysocka J. (2023). Structural elements promote architectural stripe formation and facilitate ultra-long-range gene regulation at a human disease locus. Mol. Cell.

[163] Hansen A.S., Cattoglio C., Darzacq X., Tjian R. (2018). Recent evidence that TADs and chromatin loops are dynamic structures. Nucleus.

[164] Levo M., Raimundo J., Bing X.Y., Sisco Z., Batut P.J., Ryabichko S., Gregor T., Levine M.S. (2022). Transcriptional coupling of distant regulatory genes in living embryos. Nature.

[165] Batut P.J., Bing X.Y., Sisco Z., Raimundo J., Levo M., Levine M.S. (2022). Genome organization controls transcriptional dynamics during development. Science.

[166] Paliou C., Guckelberger P., Schöpflin R., Heinrich V., Esposito A., Chiariello A.M., Bianco S., Annunziatella C., Helmuth J., Haas S. (2019). Preformed chromatin topology assists transcriptional robustness of Shh during limb development. Proc. Natl. Acad. Sci. USA.

[167] Bonev B., Cohen N.M., Szabo Q., Fritsch L., Papadopoulos G.L., Lubling Y., Xu X., Lv X., Hugnot J.P., Tanay A. (2017). Multiscale 3D Genome Rewiring during Mouse Neural Development. Cell.

[168] Li X., Levine M. (2024). What are tethering elements?. Curr. Opin. Genet. Dev..

[169] Nicodemi M., Prisco A. (2009). Thermodynamic Pathways to Genome Spatial Organization in the Cell Nucleus. Biophys. J..

[170] Barbieri M., Chotalia M., Fraser J., Lavitas L.M., Dostie J., Pombo A., Nicodemi M. (2012). Complexity of chromatin folding is captured by the strings and binders switch model. Proc. Natl. Acad. Sci. USA.

[171] Clark D.J., Kimura T. (1990). Electrostatic mechanism of chromatin folding. J. Mol. Biol..

[172] Lipfert J., Doniach S., Das R., Herschlag D. (2014). Understanding Nucleic Acid—Ion Interactions. Annu. Rev. Biochem..

[173] Simonson T., Brooks C.L. (1996). Charge Screening and the Dielectric Constant of Proteins: Insights from Molecular Dynamics. J. Am. Chem. Soc..

[174] Yokoshi M., Segawa K., Fukaya T. (2020). Visualizing the Role of Boundary Elements in Enhancer-Promoter Communication. Mol. Cell.

[175] Benabdallah N.S., Williamson I., Illingworth R.S., Kane L., Boyle S., Sengupta D., Grimes G.R., Therizols P., Bickmore W.A. (2019). Decreased Enhancer-Promoter Proximity Accompanying Enhancer Activation. Mol. Cell.

[176] Shaner N.C., Steinbach P.A., Tsien R.Y. (2005). A guide to choosing fluorescent proteins. Nat. Methods.

[177] Chudakov D.M., Matz M.V., Lukyanov S., Lukyanov K.A. (2010). Fluorescent Proteins and Their Applications in Imaging Living Cells and Tissues. Physiol. Rev..

[178] Rodriguez E.A., Campbell R.E., Lin J.Y., Lin M.Z., Miyawaki A., Palmer A.E., Shu X., Zhang J., Tsien R.Y. (2017). The Growing and Glowing Toolbox of Fluorescent and Photoactive Proteins. Trends Biochem. Sci..

[179] Grimm J.B., Muthusamy A.K., Liang Y., Brown T.A., Lemon W.C., Patel R., Lu R., Macklin J.J., Keller P.J., Ji N. (2017). A general method to fine-tune fluorophores for live-cell and in vivo imaging. Nat. Methods.

[180] Stagge F., Mitronova G.Y., Belov V.N., Wurm C.A., Jakobs S. (2013). Snap-, CLIP- and Halo-Tag Labelling of Budding Yeast Cells. PLoS ONE.

[181] Boersma S., Khuperkar D., Verhagen B.M., Sonneveld S., Grimm J.B., Lavis L.D., Tanenbaum M.E. (2019). Multi-Color Single-Molecule Imaging Uncovers Extensive Heterogeneity in mRNA Decoding. Cell.

[182] Wang L., Frei M.S., Salim A., Johnsson K. (2019). Small-Molecule Fluorescent Probes for Live-Cell Super-Resolution Microscopy. J. Am. Chem. Soc..

[183] Pradhan S., Apaydin S., Bucevičius J., Gerasimaitė R., Kostiuk G., Lukinavičius G. (2023). Sequence-specific DNA labelling for fluorescence microscopy. Biosens. Bioelectron..

[184] Lu S., Hou Y., Zhang X.E., Gao Y. (2023). Live cell imaging of DNA and RNA with fluorescent signal amplification and background reduction techniques. Front. Cell Dev. Biol..

[185] Pichon X., Robert M.C., Bertrand E., Singer R.H., Tutucci E. (2020). RNA Tagging, Methods and Protocols. Methods Mol. Biol..

[186] Durrieu L., Kirrmaier D., Schneidt T., Kats I., Raghavan S., Hufnagel L., Saunders T.E., Knop M. (2018). Bicoid gradient formation mechanism and dynamics revealed by protein lifetime analysis. Mol. Syst. Biol..

[187] Los G.V., Encell L.P., McDougall M.G., Hartzell D.D., Karassina N., Zimprich C., Wood M.G., Learish R., Ohana R.F., Urh M. (2008). HaloTag: A Novel Protein Labeling Technology for Cell Imaging and Protein Analysis. ACS Chem. Biol..

[188] Grimm J.B., English B.P., Chen J., Slaughter J.P., Zhang Z., Revyakin A., Patel R., Macklin J.J., Normanno D., Singer R.H. (2015). A general method to improve fluorophores for live-cell and single-molecule microscopy. Nat. Methods.

[189] Chen B.C., Legant W.R., Wang K., Shao L., Milkie D.E., Davidson M.W., Janetopoulos C., Wu X.S., Hammer J.A., Liu Z. (2014). Lattice light-sheet microscopy: Imaging molecules to embryos at high spatiotemporal resolution. Science.

[190] Schermelleh L., Ferrand A., Huser T., Eggeling C., Sauer M., Biehlmaier O., Drummen G.P.C. (2019). Super-resolution microscopy demystified. Nat. Cell Biol..

[191] Mertz J., Kim J. (2010). Scanning light-sheet microscopy in the whole mouse brain with HiLo background rejection. J. Biomed. Opt..

[192] Dunsby C. (2008). Optically sectioned imaging by oblique plane microscopy. Opt. Express.

[193] Stelzer E.H.K., Strobl F., Chang B.J., Preusser F., Preibisch S., McDole K., Fiolka R. (2021). Light sheet fluorescence microscopy. Nat. Rev. Methods Prim..

[194] Betzig E., Patterson G.H., Sougrat R., Lindwasser O.W., Olenych S., Bonifacino J.S., Davidson M.W., Lippincott-Schwartz J., Hess H.F. (2006). Imaging Intracellular Fluorescent Proteins at Nanometer Resolution. Science.

[195] Huff J. (2015). The Airyscan detector from ZEISS: Confocal imaging with improved signal-to-noise ratio and super-resolution. Nat. Methods.

[196] Benninger R.K., Piston D.W. (2013). Two-Photon Excitation Microscopy for the Study of Living Cells and Tissues. Curr. Protoc. Cell Biol..

[197] Becker W., Su B., Holub O., Weisshart K. (2011). FLIM and FCS detection in laser-scanning microscopes: Increased efficiency by GaAsP hybrid detectors. Microsc. Res. Tech..

[198] Luu P., Fraser S.E., Schneider F. (2024). More than double the fun with two-photon excitation microscopy. Commun. Biol..

[199] Engelbrecht C.J., Stelzer E.H. (2006). Resolution enhancement in a light-sheet-based microscope (SPIM). Opt. Lett..

[200] Chen B., Chang B.J., Roudot P., Zhou F., Sapoznik E., Marlar-Pavey M., Hayes J.B., Brown P.T., Zeng C.W., Lambert T. (2022). Resolution doubling in light-sheet microscopy via oblique plane structured illumination. Nat. Methods.

[201] Mir M., Stadler M.R., Ortiz S.A., Hannon C.E., Harrison M.M., Darzacq X., Eisen M.B. (2018). Dynamic multifactor hubs interact transiently with sites of active transcription in Drosophila embryos. eLife.

[202] Elson E. (2011). Fluorescence Correlation Spectroscopy: Past, Present, Future. Biophys. J..

[203] Athilingam T., Nelanuthala A.V.S., Breen C., Karedla N., Fritzsche M., Wohland T., Saunders T.E. (2024). Long-range formation of the Bicoid gradient requires multiple dynamic modes that spatially vary across the embryo. Development.

[204] Axelrod D., Koppel D., Schlessinger J., Elson E., Webb W. (1976). Mobility measurement by analysis of fluorescence photobleaching recovery kinetics. Biophys. J..

[205] Elf J., Li G.W., Xie X.S. (2007). Probing Transcription Factor Dynamics at the Single-Molecule Level in a Living Cell. Science.

[206] Mazzocca M., Fillot T., Loffreda A., Gnani D., Mazza D. (2021). The needle and the haystack: Single molecule tracking to probe the transcription factor search in eukaryotes. Biochem. Soc. Trans..

[207] Lelek M., Gyparaki M.T., Beliu G., Schueder F., Griffié J., Manley S., Jungmann R., Sauer M., Lakadamyali M., Zimmer C. (2021). Single-molecule localization microscopy. Nat. Rev. Methods Prim..

[208] Dey B., Thukral S., Krishnan S., Chakrobarty M., Gupta S., Manghani C., Rani V. (2012). DNA—Protein interactions: Methods for detection and analysis. Mol. Cell. Biochem..

[209] Rengachari S., Schilbach S., Aibara S., Dienemann C., Cramer P. (2021). Structure of the human Mediator—RNA polymerase II pre-initiation complex. Nature.

[210] Grandi F.C., Modi H., Kampman L., Corces M.R. (2022). Chromatin accessibility profiling by ATAC-seq. Nat. Protoc..

[211] Sati S., Cavalli G. (2017). Chromosome conformation capture technologies and their impact in understanding genome function. Chromosoma.

